# Measuring cerebral perfusion with [^11^C]-PiB R1 in Down syndrome: associations with amyloid burden and longitudinal cognitive decline

**DOI:** 10.1093/braincomms/fcaa198

**Published:** 2020-11-18

**Authors:** Elijah Mak, Monika Grigorova, Jessica Beresford-Webb, Maura Malpetti, Madeline Walpert, Stephanie Brown, Elizabeth Jones, Isabel Clare, Young T Hong, Tim D Fryer, Jonathan P Coles, Franklin I Aigbirhio, David K Menon, Peter J Nestor, Anthony J Holland, Shahid H Zaman

**Affiliations:** 1 Department of Psychiatry, University of Cambridge, School of Clinical Medicine, Cambridge Biomedical Campus, CB2 0QQ, UK; 2 Department of Clinical Neurosciences, University of Cambridge, Cambridge Biomedical Campus, Cambridge, CB2 0SZ, UK; 3 Wolfson Brain Imaging Centre, University of Cambridge, CB2 0QQ, UK; 4 Division of Anaesthesia, University of Cambridge, Cambridge, CB2 0QQ, UK; 5 Queensland Brain Institute, University of Queensland, Queensland, QLD 4072, Australia

**Keywords:** Down syndrome, Alzheimer’s disease, positron emission tomography, neurodegeneration, perfusion

## Abstract

Positron emission tomography imaging of glucose hypometabolism and amyloid deposition are two well-established methods to evaluate preclinical changes in Alzheimer’s disease and people with Down syndrome. However, the use of both imaging modalities may overburden participants, particularly those with intellectual disabilities and cognitive impairment. The relative tracer delivery of the [^11^C]-Pittsburgh Compound B has been proposed as a viable surrogate for cerebral perfusion. Here, we studied the impact of amyloid pathology on perfusion changes in Down syndrome and evaluated its associations with cognitive impairment. In total, 47 adults with Down syndrome underwent the [^11^C]-Pittsburgh Compound B imaging and structural imaging. The structural data were processed with Freesurfer to obtain anatomical segmentations and cortical thickness. The relative tracer delivery from [^11^C]-Pittsburgh Compound B was derived using a simplified reference tissue model. The sample was stratified into those with minimal amyloid burden (*n* = 25) and those with elevated amyloid (*n* = 22). We found significant and widespread reductions of cerebral perfusion in those with elevated amyloid burden, independent of age, gender, cognitive function and cortical thickness. In addition, cerebral perfusion was associated with the cognitive impairment among the Down syndrome group with elevated amyloid burden. These findings highlight the promising utility of the relative tracer delivery of the [^11^C]-Pittsburgh Compound B as a surrogate index in clinical trials for monitoring disease progression or tracking physiologic changes over time in Down syndrome.

## Teaser

Mak *et al.* showed that elevated amyloid burden among people with Down syndrome is associated with reduced cerebral perfusion. Perfusion deficits were independent of grey-matter atrophy and also correlated with worse cognitive performance. These findings demonstrate the potential utility of perfusion imaging as a surrogate marker of disease progression.

## Graphical Abstract

**Graphical Abstract:**
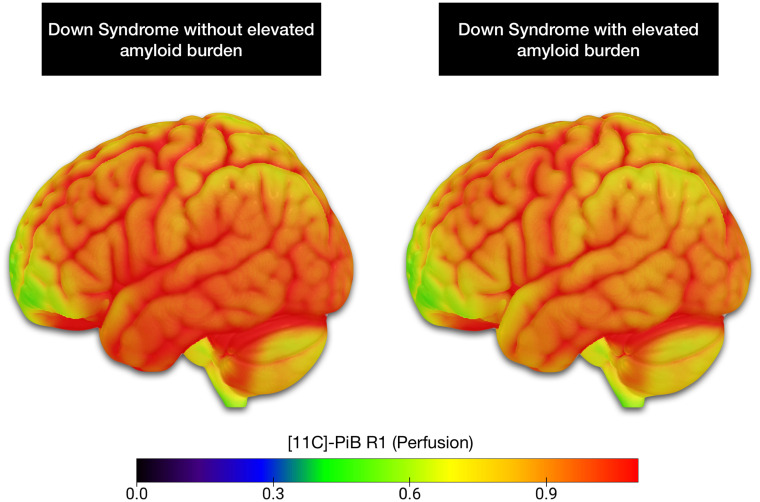

## Introduction

Down syndrome is the most common neurodevelopmental disorder caused by the presence of trisomy 21 (1:800 to 1:1000 live births worldwide). The extra copy of chromosome 21 is associated with a four- to five-fold overexpression of the amyloid precursor protein (APP) gene and increased accumulation of cerebral beta-amyloid (Aβ) deposition in the brain and subsequent neurofibrillary tau tangle formation and neurodegeneration (Wiseman *et al.*, 2015). Consequently, dementia occurs in ∼10–25% of persons with Down syndrome in their 40 years, 20–50% of those in their 50 years and 60–75% of those over the age of 60 years. Indeed, the strong dependency of Alzheimer’s disease (AD) progression on ageing also means that multi-modal neuroimaging studies in Down syndrome may help delineate the natural history of biomarker change, and in the process identify early biomarkers of AD pathogenesis that may not be logistically feasible in the general population (Neale *et al.*, 2018).

Models of AD pathophysiology propose a sequential progression of brain changes that are reflected by neuroimaging abnormalities, beginning with an early increase in Aβ PETtracer binding, followed by a gradual progression of neurofibrillary tau tangles, deficits in cerebral glucose metabolism **(**i.e. [^18^F]-fluorodeoxyglucose-PET (FDG-PET) and finally grey-matter atrophy as seen with structural T1-MRI. To this end, two of the best-established methods for evaluating the preclinical phases of AD are PET imaging of Aβ accumulation and cerebral glucose hypometabolism, both of which occur up to 25 and 10 years prior to symptom onset (Bateman *et al.*, 2012; Jack and Holtzman, 2013). The spatial topography and clinical implications of Aβ has been extensively studied in Down syndrome with PET ligands that selectively bind to Aβ (Hartley *et al.*, 2014; Jennings *et al.*, 2015; Annus *et al.*, 2017; Lao *et al.*, 2017a). Elevated amyloid is typically observed after 35 years of age (Annus *et al.*, 2016) and is associated with early cognitive impairment and brain atrophy (Annus *et al.*, 2017; Mak *et al.*, 2019b).

As a ‘state’ biomarker, Aβ PET imaging has allowed us to stratify into ‘Aβ groupings’ that aid in patient selection and enrichment of study samples; however, it lacks ability to track dementia severity in AD (Engler *et al.*, 2006). This was also illustrated in a case study of a Down syndrome patient with three serial PET scans, showing that dementia onset occurred almost 2 years after a sharp spike in Aβ burden (Mak *et al.*, 2019a). Additional FDG-PET or cerebral blood flow imaging is highly sensitive to downstream phenomenon of synaptic dysfunction and cerebral perfusion, both of which are tightly coupled and believed to reflect local neuronal dysfunction (Joseph-Mathurin *et al.*, 2018) and therefore cognitive symptoms. In sporadic AD, a characteristic pattern of glucose hypometabolism emerges involving posterior cingulate at the MCI stage, with extension to lateral temporo-parietal regions by the time mild dementia is evident (Minoshima *et al.*, 1997; Nestor *et al.*, 2003), and ultimately affecting most cortical tissue. Significant deficits in glucose metabolism are typically found in genetically at-risk individuals (Protas *et al.*, 2013) and is associated with clinical decline from MCI to AD (Drzezga *et al.*, 2003). However, few studies have investigated levels of glucose metabolism in Down syndrome, yielding mixed findings across small samples that did not account for amyloid status (Schapiro *et al.*, 1990; Lao *et al.*, 2017b).

The scarcity of studies on glucose metabolism relative to Aβ accumulation and cortical atrophy is most likely due to the prohibitive study costs and cumulative radiation exposure inherent in multi-PET tracer study designs (i.e. [^11^C]-PiB [Pittsburgh Compound B] and [^18^F]-FDG PET imaging). The issue of participant overburden is also pertinent in study samples of participants with intellectual disabilities. To circumvent these obstacles, several groups have successfully validated the relative tracer delivery (*R*_1_) of [^11^C]-PiB as a proxy of cerebral blood flow (Meyer *et al.*, 2011; Chen *et al.*, 2015; Joseph-Mathurin *et al.*, 2018), a process that is tightly coupled with glucose metabolism (Paulson *et al.*, 2010). In addition, a study from the Dominantly Inherent Alzheimer Network demonstrated that PiB-R_1_ was spatially congruent with FDG-PET and sensitive to longitudinal changes among familial mutation carriers (Joseph-Mathurin *et al.*, 2018).

To our knowledge, Aβ-associated changes in PiB-R1 and their association with clinical outcomes have not been investigated in Down syndrome, to date. Here, we studied the impact of Aβ pathology on the PiB-R1 in Down syndrome to evaluate their utility in clinical trials and further evaluate the associations of PiB-R1 with cognitive impairment. We hypothesized that (i) Down syndrome adults with elevated Aβ burden will show reductions in cerebral perfusion relative to Down syndrome adults without Aβ burden; (ii) R1 perfusion will be strongly associated with cognitive function and (iii) baseline reductions in R1 perfusion will be associated with subsequent decline in cognitive status.

## Materials and methods

### Study design and participants

In total, 47 adults with Down syndrome underwent [^11^C]-PiB-PET imaging and structural MRI. Participants were identified via services for people with intellectual disabilities in England and Scotland, through the Down’s Syndrome Association or following responses to our website. All participants had previously received a clinical diagnosis of Down syndrome based on having the characteristic phenotype. The study was approved by the National Research Ethics Committee of East of England and the Administration of Radioactive Substances Advisory Committee. Written consent was obtained from all adults with Down syndrome with the capacity to consent. For participants lacking the capacity to consent, the procedures set out in the England and Wales Mental Capacity Act (2005) or the Adults with incapacity (Scotland Act), depending on place of residence, were followed.

### Clinical assessments

All participants were assessed for dementia using the Cambridge Examination for Mental Disorders of Older people with Down’s Syndrome and Others with Intellectual Disabilities (CAMDEX-DS) informant interview, designed for diagnosing dementia in this population (Roth *et al.*, 1986). An experienced clinician (S.H.Z. or A.J.H.), who was blinded to the age of the participant and the PiB status, allocated each participant into the categories of (i) those without acquired cognitive impairment, (ii) mild cognitive decline and (iii) or dementia. Dementia was diagnosed in accordance with the International Classification of Diseases-10 (ICD-10) criteria for dementia. The diagnosis of functional ‘cognitive decline’ was given to participants with informant reported evidence of decline in one or more cognitive domains without fulfilling the full ICD-10 criteria for dementia. Thirty Down syndrome participants returned after 2–5 years for a repeat diagnosis of cognitive status. Nine Down syndrome adults experienced a progressive decline in their cognitive status **(**i.e. No acquired cognitive decline _**→**_ MCI or dementia; MCI _**→**_ dementia), whereas 21 remained cognitively stable.

### Imaging protocol

#### Structural MRI

The T1-magnetization prepared rapid gradient echo data were processed using Freesurfer to obtain cortical segmentations and region of interest (ROI) estimates of cortical thickness based on the Desikan–Killiany parcellation scheme (Desikan *et al.*, 2006). The technical details for the quantification of cortical thickness have been extensively described earlier (Fischl and Dale, 2000).

#### [^11^C]-PiB imaging

[^11^C]-PiB data were acquired in three-dimensional (3D) mode on a GE Advance scanner. Before [^11^C]-PiB injection, a 15-min transmission scan using rotating ^68^Ge rod sources was acquired to correct for photon attenuation. [^11^C]-PiB was produced with high radiochemical purity (>95%) and specific activity (>150 GBq/umol). [^11^C]-PiB was injected as a bolus (median = 545 MBq, interquartile range = 465–576 MBq) through an antecubital venous catheter, and data were acquired for 90 min after injection in 58 frames (18 × 5, 6 × 15 s, 10 × 30 s, 7 × 1 min, 4 × 2.5 min and 13 × 5 min). For each frame, sonogram data were reconstructed using the PROMIS 3D filtered back projection algorithm into a 128 × 128 × 35 image array with a voxel size of 2.34 × 2.34 × 4.25 mm^3^. Corrections were applied for random coincidences, dead time, normalization, scatter, attenuation and sensitivity. The dynamic PET images were realigned with statistical parametric mapping and then averaged. The resultant mean images were rigidly co-registered with advanced normalization tools to their corresponding bias-corrected magnetization-prepared rapid gradient echo MRI volume. Compositions of concatenated transformations (from PET native space to study template) were calculated and applied to PET images followed by linear interpolation. The intersection of the standardized Brodmannn atlas with ≥65% grey-matter probability mask was applied to spatially normalize PET images to extract time activity curves for each region, which were then subjected to reference tissue input kinetic modelling. The reference tissue ROI was the superior cerebellar region constrained to >90% grey-matter probability. To ameliorate partial volume error from CSF contamination, Gaussian smoothing was applied to the CSF segment to approximate the PET spatial resolution and hence each ROI time activity curve was divided by 1 – *f*s_CSF_, where *f*_CSF_ is the average CSF fraction in the ROI. For each ROI, BP_ND_ was obtained using a basis function implementation of the simplified reference tissue model. Pittsburgh Compound B-positive and PiB-negative groups were assigned on the basis of striatal BP_ND_, which had previously revealed a bimodal distribution with clear separation of positive (Down syndrome persons with elevated amyloid deposition, DS-POS) and negative (Down syndrome persons with minimal amyloid deposition, DS-NEG) groups (Annus *et al.*, 2016).

#### Quantification of relative delivery ratio R1 parametric images

*R*1 from PiB were derived from the full PiB dynamic time activity curve using a simplified reference tissue model on a regional basis to characterize regional perfusion relative to the cerebellar grey matter. The original and fitted time activity curve for regions with high and low amyloid burden are shown in Supplementary Fig. 1. Subsequently, the *R*1 data sets were co-registered to the structural MRI. Inverse transformations were applied to resample the Freesurfer cortical segmentations into the native *R*1 space in order to estimate regional *R*1 values.

### Statistical analyses

All statistical analyses were performed using the *R* statistical package. Normality of the main imaging measures (i.e. cortical R1, cortical PiB and cortical thickness) was assessed with skewness tests and visual inspection of density plots. Owing to the skewed distributions, mean *R*1, PiB and cortical thickness values were subjected to inverse normal transformations so as to fulfil the normality assumptions of linear regression and ANOVA (Ganjgahi *et al.*, 2015; Raffield *et al.*, 2015; Hodgson *et al.*, 2017; Tynkkynen *et al.*, 2018). Robust linear regressions were used to compare cortical thickness and *R*1 between DS-POS and DS-NEG. The model included nuisance covariates such as age, gender and cognitive status (i.e. those without acquired cognitive impairment, those with mild cognitive decline and those with dementia). A second model further adjusted for mean cortical thickness to determine whether any R1 changes are independent of brain atrophy in DS-POS. To delineate the topography of Aβ-associated R1 changes, between-group differences in regional *R*1 values were compared between the DS-POS and DS-NEG groups, adjusting for age, gender and cognitive statuses. False-discovery rate correction was performed to account for multiple tests across the 68 cortical regions. The next set of analyses was designed to address our hypothesis that cortical *R*1 is associated with cognitive function. Among the DS-POS group with cognitive impairment, we used partial Spearman rank correlation to test the association between CAMCOG total scores and cortical *R*1, adjusting for age, gender and mean cortical thickness. Finally, we also compared mean cortical *R*1 between Down syndrome adults who remained cognitively stable (*n* = 21) and those who showed a decline in cognition that warranted a change in diagnosis, while adjusting for age, gender, cognitive status and PiB status.

### Data availability

Data are available upon reasonable requests.

## Results

### Clinical characteristics

Group comparisons of demographics and cognitive data are summarized in Table 1. The DS-POS group was significantly older and cognitively impaired relative to the DS-NEG group. Gender distribution was not significantly different between groups.

**Table 1:** Demographics and clinical characteristics of the study sample

	DS-NEG (*n* = 25)	DS-POS (*n* = 22)	*P* value
Age	37.8 ± 5.7	48.1 ± 7.5	<0.001
Gender	60%	45%	0.32
CAMCOG	78.6 ± 16.4	61.9 ± 24.6	0.04
Cognitively intact	88%	50%	0.02

DS-NEG, amyloid-negative Down syndrome adults; DS-POS, amyloid-positive Down syndrome adults; CAMCOG, Cambridge Cognitive Examination.

### Group comparisons of mean cortical *R*1 and thickness

#### Global comparisons

After adjusting for age, gender and cognitive status, robust linear regressions showed that DS-POS adults had significantly reduced mean cortical *R*1 compared to DS-NEG adults (*P* = 0.01). The Wilcoxon Rank Sum test was also used to evaluate group differences of mean *R*1, showing significantly decreased mean *R*1 in DS-POS relative to DS-NEG (*W* = 481, *P* _**<**_ 0.001). Furthermore, the *R*1 deficit remained significant after controlling for mean cortical thickness (*P* = 0.03). In contrast, there was only a trend-level difference in cortical thickness within the model including age, gender, cognitive status and mean cortical *R*1 as covariates (*P* = 0.085). A supplementary receiver operator characteristics analysis showed that age-adjusted *R*1 values achieved an area under the curve of 64% in separating DS-POS from DS-NEG cases (Supplementary Fig. 2).

#### Regional comparisons

After adjusting for age, gender and cognitive status, we observed a widespread pattern of significant *R*1 reductions in the DS-POS group, predominantly in the temporo-parietal cortices and frontal cortices (false-discovery rate, *P* < 0.05, Fig. 2). Including the mean cortical thickness or local cortical thickness did not substantially affect the findings.

**Figure 1:**
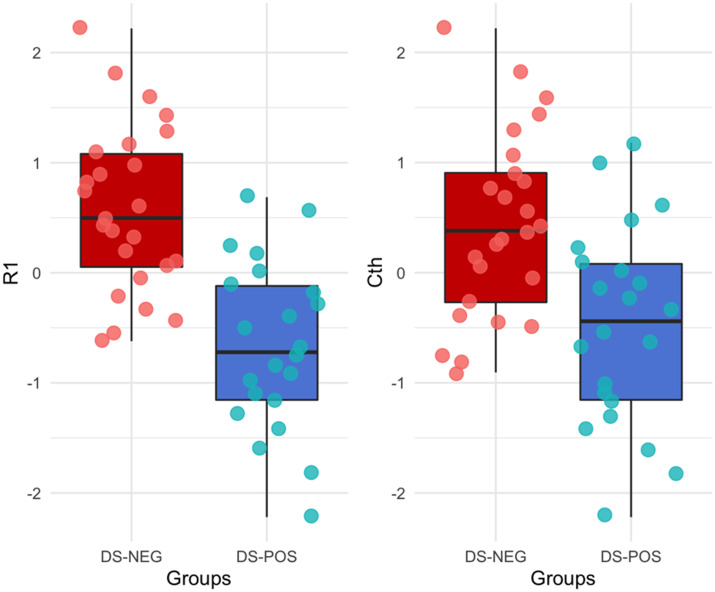
**Boxplots of rank transformed mean cortical *R*1 and cortical thickness values in each Down syndrome amyloid group.** After adjusting for age, gender and cognitive status and mean cortical thickness, DS-POS adults showed significantly reduced mean *R*1 compared to DS-NEG adults. DS-NEG, amyloid-negative Down syndrome adults; DS-POS, amyloid-positive Down syndrome adults; *R*1, relative influx of the [^11^C]-PiB tracer; Cth, cortical thickness.

**Figure 2:**
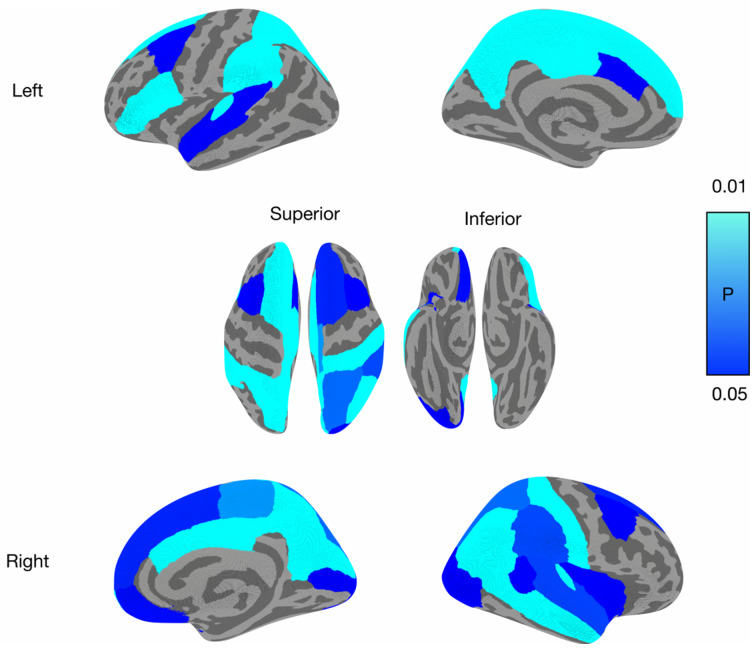
**Decreased regional *R*1 in DS-POS relative to DS-NEG adults.** Regional FDR-adjusted *P* values of reduced *R*1 are overlaid on each of the cortical regions. Brighter colours reflect a greater magnitude of difference. Covariates include age, gender and cognitive status. DS-NEG, amyloid-negative Down syndrome adults; DS-POS, amyloid-positive Down syndrome adults; *R*1, relative influx of the [^11^C]-PiB tracer; FDR, false-discovery rate.

### Relationship between perfusion and cognitive function

**Figure 3:**
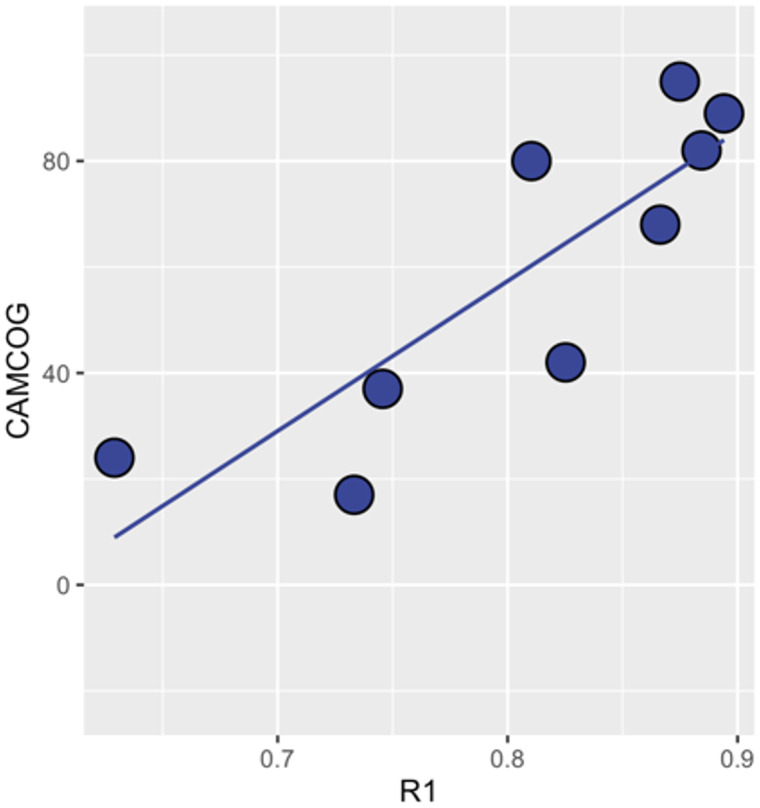
**Association between cortical perfusion and CAMCOG scores among DS-POS individuals with cognitive impairment.** *R*1, relative influx of the [^11^C]-PiB tracer; CAMCOG, Cambridge Cognitive Examination.

Among DS-POS cases with cognitive impairment, partial spearman correlations indicated a significant and positive association between mean cortical *R*1 and total CAMCOG after adjusting for age, gender and mean cortical thickness (Spearman’s *R* = 0.84, *P* = 0.037; Fig. 3). In addition, there was a significant PiB Status by *R*1 interaction in our robust linear regression models, suggesting that the effect of *R*1 in predicting CAMCOG scores was significantly higher in the DS-POS group compared to the DS-NEG group (*P* = 0.033, adjusted for age, gender, cognitive status and mean cortical thickness).

### Cerebral perfusion at baseline-predicted subsequent cognitive decline

**Figure 4:**
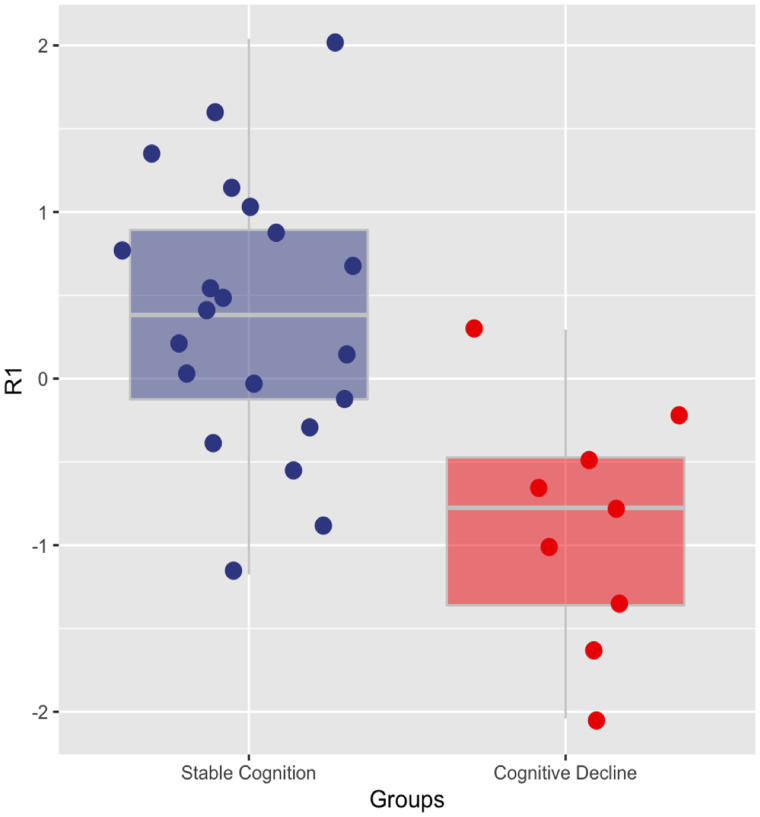
**Comparison of cortical *R*1 between Down syndrome adults who remained cognitively stable and DS adults with subsequent cognitive decline.** Robust linear regression indicated significantly reduced cortical *R*1 in Down syndrome adults who subsequently showed cognitive decline compared to Down syndrome adults who remained cognitively stable. DS-NEG, amyloid-negative Down syndrome adults; DS-POS, amyloid-positive Down syndrome adults; *R*1, relative influx of the [^11^C]-PiB tracer.

Between 2010 and 2018, nine Down syndrome adults experienced a progressive decline in their cognitive status (i.e. No acquired cognitive decline → MCI or dementia; MCI → dementia), whereas 21 remained cognitively stable. Compared to the group of Down syndrome adults who remained cognitively stable, the cognitive decliners had significantly lower *R*1 at baseline, adjusted for age, gender and cognitive status (robust linear regression *P* < 0.01, Fig. 4).

## Discussion

Imaging biomarkers that are sensitive to the earliest stages of AD progression and cognitive impairment are critical for the optimization of clinical trials. Although hypometabolism is a highly sensitive measure of neurodegeneration, [^18^F]-FDG PET imaging comes with drawbacks in terms of logistical challenges and cumulative exposure to radioactive tracers for research participants. In this study, we report the first application of [^11^C]-PiB *R*1 in a cohort of people with Down syndrome and evaluated its utility as a biomarker for use in clinical trials. In keeping with our hypotheses, this study demonstrated (i) that Aβ accumulation is accompanied by decreased cerebral perfusion prior to the onset of dementia; (ii) deficits of *R*1 in DS-POS retained statistical significance after additional adjustment for cortical thickness; (iii) baseline [^11^C]-PiB *R*1 is strongly associated with cognitive impairment and subsequent decline in cognitive status.

To the best of our knowledge, there have not been any prior reports of [^11^C]-PiB *R*1 in Down syndrome cohorts. Given the lack of [^18^F]-FDG PET in this study, it is important to interpret our [^11^C]-PiB *R*1 findings in light of the existing literature in AD and other familial forms of dementia. Relative to the DS-NEG group, the reduction of cortical perfusion in the presence of elevated Aβ burden is highly consistent with the temporal trajectory of biomarker events proposed in the amyloid cascade model (Jack *et al.*, 2013). For instance, ^18^F-fluorodeoxyglucose (FDG)-PET studies reveal characteristic and progressive metabolic reductions in the posterior cingulate, precuneus, and parietal, temporal, and prefrontal brain regions beginning years before the clinical onset of AD. Furthermore, our regional analyses revealed a topography of perfusion deficits in DS-POS that is well aligned with published data in AD and autosomal dominant AD (ADAD) (Joseph-Mathurin *et al.*, 2018). In particular, the DS-POS group showed significant perfusion deficits in precuneus, posterior cingulate and lateral temoro-parietal regions—regions that are preferentially affected in the early stages of AD with FDG-PET. Our data also substantiated a similar finding of hypometabolism in a smaller sample of Down syndrome adults with a clinical diagnosis of AD (*n* = 5) (Sabbagh *et al.*, 2015). Decreased posterior cingulate metabolism has also been observed in asymptomatic APOE4 carriers in the general population (Protas *et al.*, 2013).

At present, one of the key challenges for a candidate biomarker in AD clinical trials concerns its sensitivity to track disease-related changes that occur before the overt manifestations of cognitive and functional decline. In AD, the temporal evolution of biomarkers has often been extrapolated on the basis of biomarker severity in cross-sectional designs (Jack *et al.*, 2013). To this end, the severe reductions of *R*1 perfusion seen in our DS-POS group were still apparent even after controlling for cortical thinning (as well as age, gender and cognitive status). Interestingly, disproportionate hypometabolism relative to structural atrophy has been reported in pre-symptomatic individuals carrying mutations in the Presenilin 1 gene (Mosconi *et al.*, 2006), whereas reductions of posterior cingulate [^18^F]-FDG deficits were still detected after controlling for hippocampal volumes (Protas *et al.*, 2013). Together, these findings dovetail with similar reports in in AD and MCI, where hypometabolism has been found to more severe compared to atrophy (De Santi *et al.*, 2001). As reductions in FDG-PET or R1 are understood to reflect synaptic loss or dysfunction, it is likely that neuronal injury is underway but has not reached the critical threshold to result in atrophy detectable with MRI.

An important pre-requisite for a biomarker is the extent to which it is capable of tracking disease progression and monitoring outcomes over time. To this end, we found that *R*1 perfusion was strongly associated with the CAMCOG scores among DS-POS individuals with cognitive impairment. Although others have reported relationships of glucose metabolism with cognitive measures in Down syndrome (Haier *et al.*, 2003; Sabbagh *et al.*, 2015; Matthews *et al.*, 2016), no previous study has determined whether the correlations were independent of existing Aβ status or degree of cortical thinning. Our interaction analyses also showed that amyloid status exerted a strong interaction on the coupling between *R*1 and CAMCOG scores, such that the effect of *R*1 on cognitive impairment was most pronounced when accompanied by the presence of abnormal Aβ burden. Similar evidence of Aβ status interacting with relationships between downstream processes has been reported in cognitively normal elderly groups (i.e. tau PET and hippocampal atrophy) (Wang *et al.*, 2016).

We also evaluated the potential utility of perfusion *R*1 to predict subsequent clinical deterioration. Compared to Down syndrome individuals who remained cognitively stable throughout the assessment period, those who exhibited a decline in cognitive function were found to have significantly reduced *R*1. This finding is consistent with longitudinal evidence, showing metabolic reductions preceding the onset of AD in MCI (Mosconi, 2005) and cognitively normal individuals (Mosconi *et al.*, 2009), and collectively implicate cerebral perfusion loss as an early Alzheimer-related process foreshadowing future cognitive decline; cortical *R*1 may be used alongside PiB imaging to identify clinical drug trial participants who may be likely to exhibit cognitive impairment within a short window of time. Considering the small sample size included in this comparison, future prospective studies in other independent longitudinal cohorts of Down syndrome are ultimately necessary to confirm our result.

The results herein show promising utility for the PiB-R1 to be used as a surrogate index in clinical trials for monitoring disease progression or tracking physiologic changes over time. However, several caveats should be considered. The calculation of the PiB-R1 entails a full dynamic PET scan that may not be well tolerated by individuals with cognitive impairment or other intellectual disabilities. In the interest of avoiding a full dynamic PET scan, early-PiB SUVR has been evaluated as an alternative over *R*1. However, the limitations of early PiB SUVR include (i) under-estimation of cerebral blood flow signal, (ii) weaker correlations with cognitive data and (iii) poorer discriminative ability compared to the *R*1 (Ottoy *et al.*, 2019). Our study also has several limitations. Not all Down syndrome adults returned for follow-up cognitive assessments and the stratification of Down syndrome adults into those with stable cognition (*n* = 21) and decliners (*n* = 9) resulted in unbalanced sample sizes. Diagnosing dementia in Down syndrome populations remains inherently challenging amidst the clinical background of intellectual disability, lack of information regarding premorbid level of functioning and difficulties in communication the full extent of cognitive impairment.

Down syndrome represents the largest population of individuals at risk for AD, far exceeding the number of people who are currently carrying the autosomal dominant AD mutations. In the context of better healthcare and consequently longer life spans, there is an urgent need to develop clinical trials in this vulnerable population. The findings in this study show clear utility of [^11^C]-PiB R1 in clinical research or trials. Although our findings are not intended to make an argument that PiB-R1 is a more sensitive marker of neuronal injury or synaptic dysfunction compared to [^18^F]-FDG PET, the significant reductions of *R*1 in DS-POS and their strong associations with cognitive outcome measurements suggest that [^11^C]-PiB-*R*1 may be a viable biomarker while minimizing radiation exposure, participant burden and overall study costs.

## Supplementary material

Supplementary material is available at *Brain Communications* online.

## Supplementary Material

fcaa198_Supplementary_Data

## Abbreviations

AD =: Alzheimer’s disease
APP =: amyloid precursor protein
Aβ =: beta-amyloid
CAMCOG =: Cambridge Cognitive Examination
CAMDEX-DS =: Cambridge Examination for Mental Disorders of Older people with Down’ Syndrome and Others with Intellectual Disabilities
DS =: Down syndrome
DS-NEG =: Down syndrome persons with minimal amyloid deposition
DS-POS =: Down syndrome persons with elevated amyloid deposition
FDG =: [^18^F]-fluorodeoxyglucose
ICD-10 =: International Classification of Diseases-10 criteria
MCI =: mild cognitive impairment
PiB =: Pittsburgh Compound B
R1 =: relative tracer delivery
ROI =: region of interest
