# Supplementary material for: Measuring cerebral perfusion with [11C]-PiB R1 in Down syndrome: associations with amyloid burden and longitudinal cognitive decline
Source: Brain Commun. 2020 Nov 18;3(1):fcaa198. doi: 10.1093/braincomms/fcaa198 (PMC7849981; doi:10.1093/braincomms/fcaa198)
Supplement: fcaa198_Supplementary_Data [file fcaa198_supplementary_data.pdf]

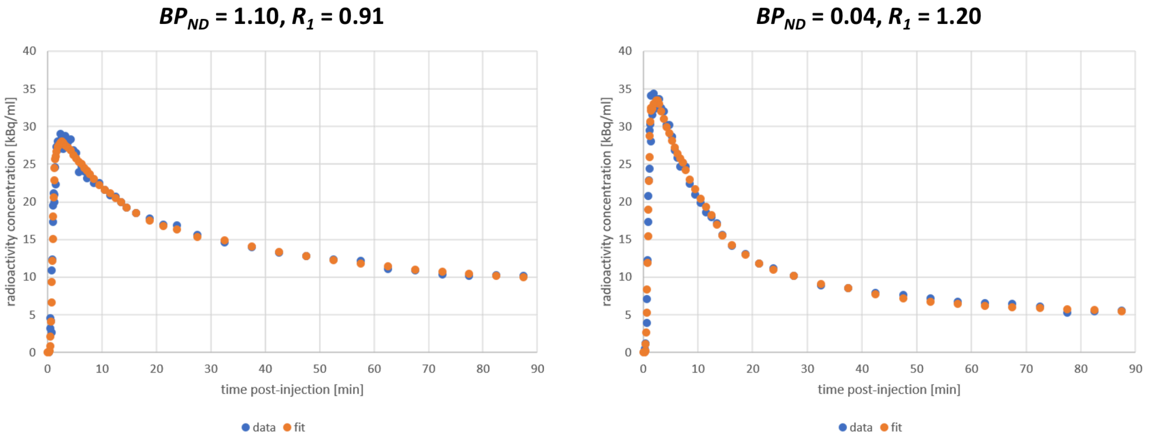

Supplementary Figure 1. Time activity curves for original and fitted data in two regions of differential amyloid burden from the same scan.

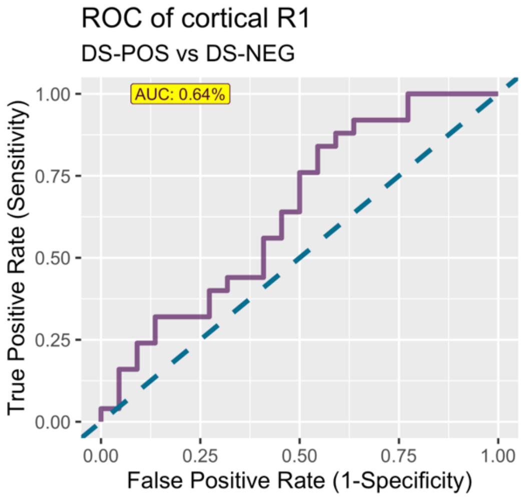

Supplementary Figure 2. Receiver Operating Characteristic analysis of cortical R1. Age-adjusted R1 achieved an AUC of 64% in discriminating DS-POS adults from DS-NEG adults. Abbreviations: DS-NEG = Amyloid-negative Down Syndrome adults; DS-POS = Amyloid-positive Down Syndrome adults; R1 = Relative influx of the [^{11}C]-PiB tracer.
